# Droplet Size Distribution in Emulsions

**DOI:** 10.1021/acs.langmuir.3c02463

**Published:** 2023-12-20

**Authors:** Manon L’Estimé, Michael Schindler, Noushine Shahidzadeh, Daniel Bonn

**Affiliations:** † Van der Waals-Zeeman Institute, Institute of Physics, University of Amsterdam, 1098XH Amsterdam, The Netherlands; ‡ CNRS UMR7083, ESPCI Paris, Université PSL, 10 Rue Vauquelin, 75005 Paris, France

## Abstract

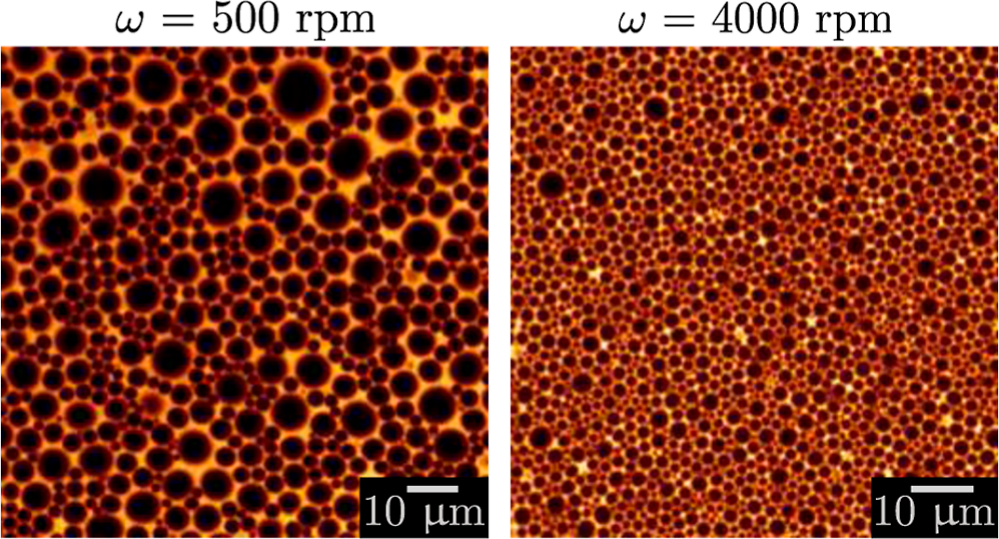

The droplet size in emulsions is known to affect the rheological properties and plays a crucial role in many applications of emulsions. Despite its importance, the underlying mechanisms governing droplet size in emulsification remain poorly understood. We investigate the average drop size and size distribution upon emulsification with a high-shear mixer for model oil-in-water emulsions stabilized by a surfactant. The size distribution is found to be a log-normal distribution resulting from the repetitive random breakup of drops. High-shear emulsification, the usual way of making emulsions, is therefore found to be very different from turbulent emulsification given by the Kolmogorov-Hinze theory, for which power-law distributions of the drop size are expected. In agreement with this, the mean droplet size does not follow a scaling with the Reynolds number of the emulsification flow but rather a capillary number scaling based on the viscosity of the continuous phase.

## Introduction

An emulsion is a dispersion of droplets in a continuous phase produced by dispersing one fluid into another immiscible fluid. The rheological properties of emulsions are of great interest, for instance, for food and cosmetic products^1,2^ and above all for the homogeneity of the flow of such materials.^3^ For concentrated emulsions, the rheology is determined by the Laplace pressure γ/*r* that indicates the deformability of individual droplets; here, γ is the interfacial tension and *r* is the droplet radius.^4−6^ Consequently, the rheology can be tuned by changing the size of the droplets, which is a key factor for the quality of the final product as it can affect its stability, flavor, texture, and mouthfeel.^7^

Since the pioneering work of Hinze and Kolmogorov on the breakup of droplets in a turbulent flow,^8,9^ numerous studies have been reported on the droplet size distribution in emulsions. A large part of the work focuses on the impact of the formulation variables, such as the viscosities of the fluids,^10−12^ the volume fraction of the dispersed phase,^13^ or the interfacial tension.^14,15^ It was shown recently that the rheology of simple emulsions can be understood on the basis of the volume fraction, interfacial tension, and drop radii, so knowing the drop radii allows us to predict the rheology^16^ if the interfacial tension is known. We therefore need to establish what governs the drop size.

Various mechanistic models have been proposed to characterize and predict the droplet size distribution. Most of these rely on the Kolmogorov–Hinze theory of turbulent emulsification in which the breakup mechanisms depend on the Kolmogorov length scale given by the size of the smallest eddies. Droplets larger than this length will break up under the action of turbulent inertial stress induced by the pressure fluctuations along the drop surface; the smaller ones remain intact. At scales smaller than the Kolmogorov length scale, cohesive forces resulting from the interfacial tension and drop viscosity oppose drop fragmentation. Hence, the maximal stable droplet diameter results from the equilibrium between the internal cohesive and external turbulent stresses, and the drop size therefore depends on the Reynolds number (or energy dissipation rate). In simulations, turbulent emulsification has been studied in detail recently,^17,18^ for which power-law distributions of the drop size were found to be in agreement with the idea that the turbulent energy cascade is important for drop formation.^18^ However, also log–normal and Gamma function drop size distributions were reported,^17^ so it is unclear as to what determines the size distribution. In an extensive study, Vankova et al.^19^ showed that although the average drop size is well described by the Kolmogorov–Hinze theory, the drop size distribution is well fitted by a log–normal distribution that does not follow from any turbulent emulsification theory. In addition, while this theory identifies a clear emulsification mechanism, it assumes a homogeneous and isotropic turbulence that is hardly achieved during the process.^20^ The presence of multiple phases will affect the turbulence itself, as known from turbulent drag reduction.^21^ In dense emulsions, it is impossible to separate the flow of the continuous phase from the motion and deformation of the discrete phase.

Alternatives to the turbulent emulsification theories are fragmentation theories, which describe the breakup due to either surface tension or the drag with the continuous phase, without necessarily requiring a turbulent flow. There are two competing fragmentation theories for the droplet size distribution. First, the breakup of droplets can be viewed as a sequence of random multiplicative processes resulting in a drop size distribution that is well described by a log–normal distribution^22,23^
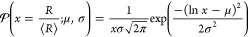
1

Second, one may have liquid threads (“ligaments”) forming through the Kelvin-Helmholtz instability that subsequently break up into droplets due to the surface tension. As demonstrated by Villermaux et al.,^24^ the breakup of ligaments leads to a Gamma distribution of sizes, namely,
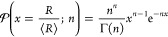
2where Γ is the gamma function and *n* is a parameter that depends on the ligament corrugation.

Also, the contrary of fragmentation is possible: the coalescence of small droplets into large ones. In principle, both processes may happen, but their rates are very different. The main reason for this asymmetry is the presence of surfactants, which hinder coalescence. As a more subtle mechanism, we have no reason to assume that these rates are constants. If they depend on the current state of the flow itself and thus on the droplet size distribution, then the fragmentation follows a nonlinear process, allowing the dominance of fragmentation over coalescence.

In practical situations, mostly rotating emulsifiers such as the one used in the present study are used. For such setups, the influence of several parameters has been investigated: speed and type of impeller were looked at, as well as the location of the dispersed phase addition.^11,25^ In addition, different emulsification devices were compared,^26^ as well as the mode of operation (batch or continuous).^27^ However, no clear unified picture of the drop size and its distribution emerged from these studies.

In the present work, we investigate how the droplet size, its mean, and its distribution are both influenced by the impeller speed, by the system formulation, and by the mixer geometry. This allows us to propose a simple scaling for the mean droplet size that does not invoke the turbulent energy cascade and allows us to distinguish between the two fragmentation models, favoring the random breakup scenario.

## Materials and Methods

### Preparation of the Emulsions

**Figure 1:**
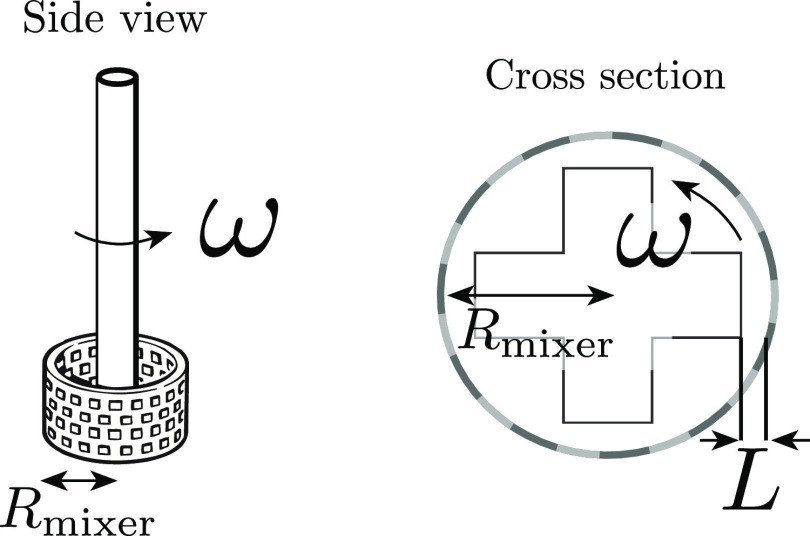
Main components of the mixer. The cross-shaped impeller rotates at a speed of ω and is surrounded by the stator. The latter is an open cylinder with a radius of *R*_mixer_ whose surface is covered by small holes. The distance between the blades and the cylinder is denoted *L*. The latter differs slightly according to the inner radius of the cylinder: *L* = 0.25 or 0.3 mm when *R*_mixer_ = 0.8 or 1.6 cm, respectively.

Oil-in-water emulsions are prepared using a Silverson high-shear rotor/stator laboratory mixer (LM5-A). As depicted in Figure 1, the mixer is composed of a rotor with a cross-shaped impeller spinning at a speed of ω. The impeller is surrounded by the stator, an open cylinder with a surface covered by small square holes. We denote by *R*_mixer_ the inner radius of the stator and by *L* the distance between the rotor extremities and the inner part of the cylinder.

We use castor oil-in-water emulsions stabilized by sodium dodecyl sulfate (SDS) surfactant as our model emulsion. The dispersed phase consists of castor oil of viscosity η_d_ = 580 mPa s. The continuous phase is prepared by dissolving 3 wt % of SDS surfactant in demineralized water. Rhodamine B is then added to the solution as a dye. As discussed later, the large concentration of surfactants inhibits droplet coalescence and ensures that the total number of surfactant molecules present is greater than the quantity needed to stabilize the emulsion.

To prepare the emulsions, we first gradually added oil to the aqueous solution while stirring at 500 rpm. Once we have reached the desired volume ratio, the rotation speed is increased step-by-step up to 4000 rpm. To homogenize the mixing, the beaker is constantly rotated around the axis of the rotor.

### Data Acquisition

**Figure 2:**
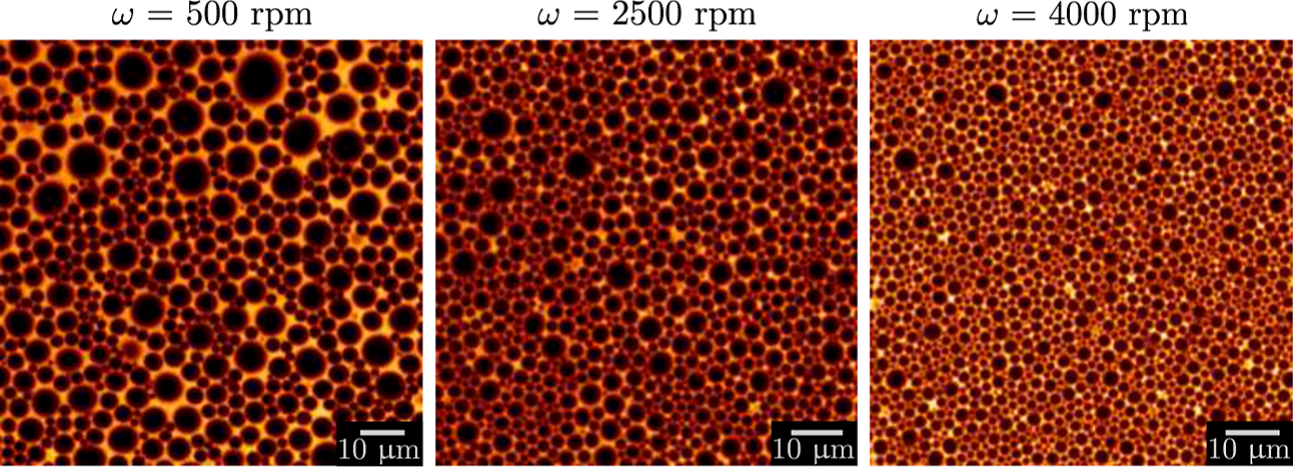
Confocal images of an emulsion at successive rotation speeds ω = 500, 2500, and 4000 rpm. The emulsion has been prepared using a mixer of radius *R*_mixer_ = 1.6 cm to disperse 80% of castor oil in an aqueous solution of viscosity η_c_ = 1 mPa s, containing 3 wt % of SDS. The oil droplets appear black in contrast with the continuous phase that contains Rhodamine B dye.

During the process, for each rotation speed, samples of the emulsion are collected and visualized by using confocal laser scanning microscopy. These pictures show circular sections through individual droplets, see Figure 2. They are then detected and measured by the ImageJ software with its plug-in “analyse particle”.^28,29^

### Data Treatment

From the observed two-dimensional (2D) circle radii, we want to conclude on the distribution of the three-dimensional (3D) spherical radii of the droplets. This challenge has several aspects, namely, (i) the question of whether and how it is possible to obtain the original 3D distribution from a distribution of 2D sections, assuming that we deal with nice smooth distribution densities. Then, (ii) the question of how to repeat the same task on a finite number of observed 2D radii.

In the following, *R* denotes the 3D radii of spheres, distributed according to a probability density ρ_R_. The radii and their distribution of 2D sections are denoted as *r* and ρ_r_, respectively.

In order to answer question (i), we assume that when an emulsion of spheres is cut by a confocal plane, any sphere of radius *R* is cut statistically independently and uniformly at height *z* in the interval [−*R*, *R*]. This implies some independence in the positions of the spheres, which are rather implicit and are beyond the scope of the present work. The combined probability density to find a sphere of radius *R* cut at height *z* is then ρ_*c*_(*R*, *z*) = ρ_*R*_(*R*)ρ(*z*|*R*), with
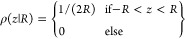
The probability density ρ_r_ of the observed cut radii *r* follows as
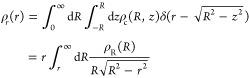
We need to invert this integral equation and obtain ρ_*R*_ from ρ_*r*_. To ease this task, we work with squared radii. The corresponding integral equation for the densities ρ_*r*^2^_ and ρ_*R*^2^_ is
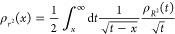
Up to the integral bounds, the inversion of this is known as Abel’s problem, and a procedure on how to solve it is given in Section 1.3.4 of ref (30). The result is
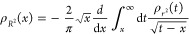
3

A critical comment on this inversion is in order: we do not fully understand in what function spaces it works. In particular, eq 3 does not guarantee ρ_*R*^2^_ to be a distribution density; it can be negative. On the other hand, it respected the total integral very well in all the cases we applied it to. We tested the method on artificially generated data and found that it works. In the measured data, we indeed found negative values in ρ_*R*^2^_. This problem was more pronounced in those data sets that do not contain very small radii.

For the numerical treatment and to answer the above question (ii), we used the best resolution on the input data, that is, ρ_*r*^2^_ being the sum of delta functions, representing the individual measured radii. The integral is then a highly irregular function of *x*, consisting of many (weak) singularities . Before taking the derivative, we smoothed this function with a Gaussian convolution kernel; its width was chosen such that the result shows well the global behavior of the curve, without too many fluctuations.

When applied to the mean radii, the above equations predict that
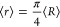
4

We tested this relation in a preliminary experiment in which we analyzed the confocal cuts of a transparent emulsion at successive heights. We used a silicone oil-in-glycerol/water emulsion stabilized by a SDS surfactant. The continuous phase is prepared by dissolving 3 wt % of SDS in a 50:50 mixture of glycerol and demineralized water. The dispersed phase consists of silicone oil of viscosity η = 500 mPa s, in which Nile red is added as a dye. Owing to the addition of glycerol to the water, the refractive indexes of the two phases are matched; thus, the emulsion is transparent. We proceed as previously to prepare an emulsion composed of 80 wt % of oil. Samples are collected at rotation speeds ω = 3000, 5000, and 7000 rpm and analyzed by confocal microscopy to measure the droplet radii at successive heights.

**Figure 3:**
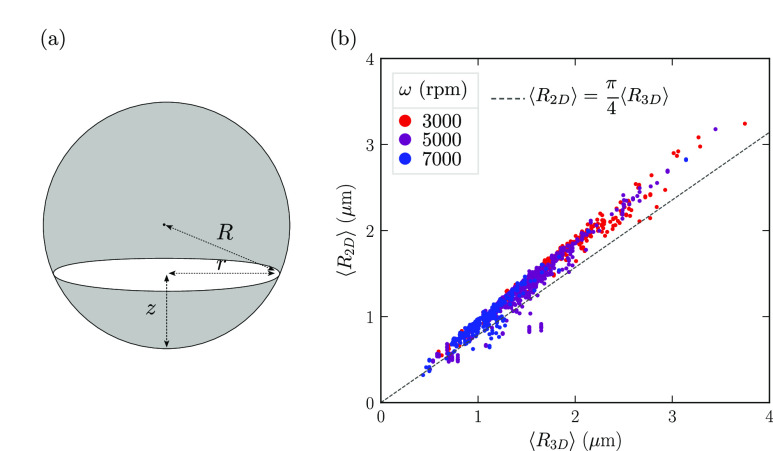
(a) The radius *r* of a sphere’s cross section at a random height *z* is likely to be smaller than the true radius *R* of the sphere. (b) Experimental determination of the correction factor by averaging the droplet’s 2D radii measured on a cross-section as a function of the average of their 3D radii, using confocal images of a transparent emulsion at different heights. The data points include three samples corresponding to rotation speeds ω of 3000 (red dots), 5000 (purple dots), and 7000 rpm (blue dots), where each dot corresponds to a cross-section. The dashed line of slope 0.79 corresponds to the theoretical correction factor derived in eq 4.

Each droplet is characterized by a set of 2D radii in which the larger one approximates the “true” 3D droplet radius. Figure 3 compares the average of the observed 2D radii, ⟨*R*_2D_⟩, with the average of the corresponding 3D radii, ⟨*R*_3D_⟩, for each cross section. The graph includes data from the three samples, each being characterized by a rotation speed ω = 3000 (red dots), 5000 (purple dots), or 7000 rpm (blue dots). The dashed line of slope 0.79 corresponds to the theoretical correction factor.

The data collapse on line with a slope of 0.91, larger than the slope predicted by the theory. This discrepancy is most likely due to the estimation of the 3D droplet radius, as the larger value of the set of 2D radii tends to slightly underestimate the true radius of the droplet. Nevertheless, the linear relationship between ⟨*R*_2D_⟩ and ⟨*R*_3D_⟩ still holds.

## Results and Discussion

In the first experiment, we demonstrate that the coalescence effects are hindered by the large concentration of surfactants. Thereafter, we vary the rotation speed ω, the oil volume fraction ϕ, the mixer geometry, and the viscosity η_*c*_ of the continuous phase to find the key ingredients determining the droplet size.

### Ensuring a Negligible Droplet Coalescence

**Figure 4:**
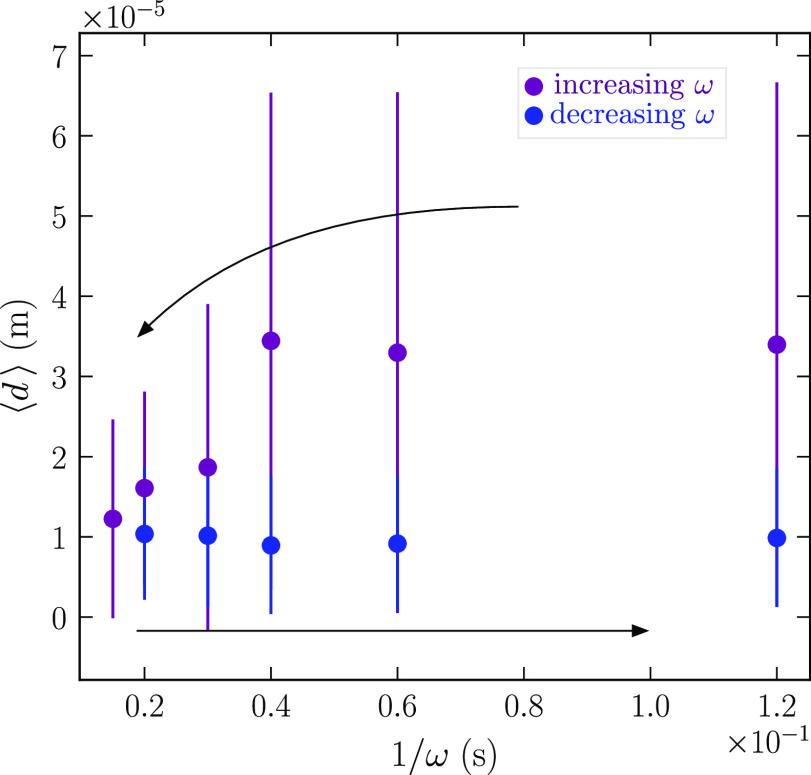
Demonstration of the absence of coalescence. The rotation speed ω is changed according to the arrow directions, leading to a hysteresis cycle of mean droplet diameter ⟨*d*⟩. The emulsion contains 3 wt % of SDS and 40 wt % of castor oil; it has been prepared with a mixer of radius *R*_mixer_ = 0.8 cm and a continuous phase of viscosity η_c_ = 1 mPa s. The vertical bars report the standard deviation.

The high concentration of surfactants in the system is expected to prevent droplet coalescence. In order to confirm this, we conducted an experiment in which the rotation speed was first increased and then decreased in a stepwise manner while samples were collected. Figure 4 shows the variation of mean droplet diameter ⟨*d*⟩ with rotation speed ω. The (upper) purple dots correspond to increasing speed (from right to left) and the (lower) blue dots correspond to decreasing speed (from left to right). The arrows indicate the succession of the data points. Initially, the increase of the impeller speed leads to a reduction in droplet size. However, during the subsequent decrease of the rotation speed, this change is not reversed, but instead, the droplets remain small. We interpret this as a result of the high concentration of surfactants effectively preventing droplet coalescence.

### Influence of the Rotation Speed

Figure 2 shows three confocal images of the same emulsion at rotation speeds ω = 500, 2500, and 4000 rpm. The dispersed oil droplets contain Rhodamine B dye and appear black. The other parameters of the emulsion are *R*_mixer_ = 1.6 cm, ϕ = 0.8, and η_c_ = 1 mPa s.

**Figure 5:**
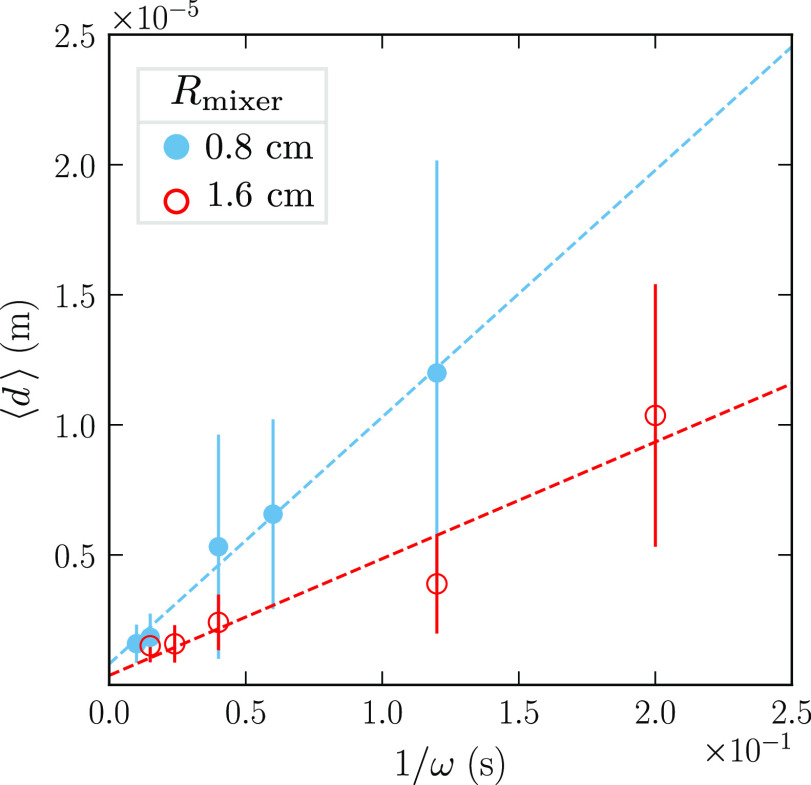
Mean droplet diameter ⟨*d*⟩ as a function of the inverse rotation speed 1/ω for emulsions prepared with ϕ = 0.8, η_c_ = 1 mPa s, and *R*_mixer_ = 0.8 cm (blue dots) or 1.6 cm (red circles). The mean droplet diameter (dots) decreases with the mixer radius and scales as the inverse of the rotation speed. The standard deviation of the diameter (vertical lines) decreases likewise.

With increasing rotation speed, the number of droplets also increases, while their size becomes smaller. The mean droplet diameter ⟨*R*⟩ is calculated for each picture and plotted against the inverse rotation speed in Figure 5 (red circles). The mean droplet diameter scales as 1/ω and decreases from 10 to 1 μm between 500 and 4000 rpm. The vertical bars indicate the standard deviation, which is rather large especially at low rotation speeds. Indeed, the complexity of such dense systems inevitably leads to some polydispersity that decreases at higher rotation speeds as shown in Figure 2.

**Figure 6:**
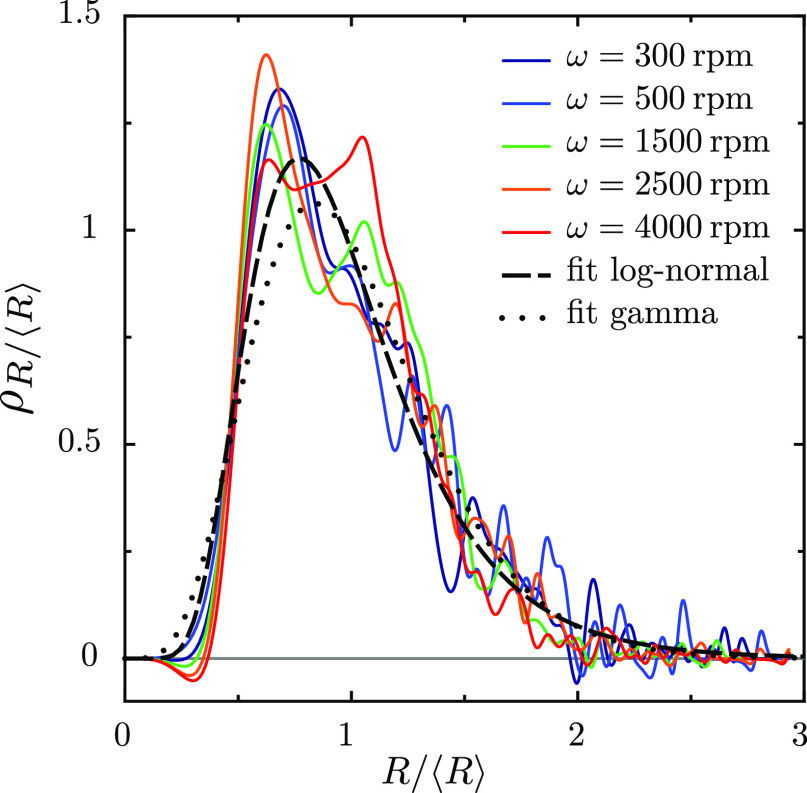
Probability densities of the droplet sizes, as obtained from the inversion eq 3, for various rotation speeds ω. The data are the same as those for *R*_mixer_ = 1.6 cm in Figure 5. While the mean droplet diameter varies with the rotation speed, the shapes of the distributions are similar. The log-normal fit has μ = −0.0957 and σ = 0.408, and the gamma fit has *n* = 6.138.

Figure 6 shows the size distributions of the sphere radii for several rotation speeds ω. The emulsions were prepared with *R*_mixer_ = 1.6 cm, ϕ = 0.8, and η_c_ = 1 mPa s (same experiments as the pictures and the red circles in Figure 5). Different rotation speeds lead to different distributions, but they collapse to a single curve when the sizes are rescaled by their average. This gives some hope that the distribution can be used to discriminate among the different theories.

We find that the data are slightly better fitted by a log-normal distribution than by a gamma distribution: the error in the fits, quantified as the summed square errors between the data and the model, behaves as 1 ÷ 1.6 (or as 1 ÷ 2.3 when fits were done to cumulative density functions instead of density functions). None of the distributions look like power-law behavior. This trend, namely, that log–normal fits slightly better than gamma was equally present when we fitted individual curves (not shown). Both fitting functions appear to underestimate the steep ascent at small values of the radius; this is perhaps due to the finite resolution of the microscope.

Within the range of fluctuations, we can say that the curves in Figure 6 are all the same. Indeed, while the family of distributions has one scale parameter and maybe several shape parameters, all curves in Figure 6 seem to have the same shape parameters. It appears therefore as a valid approximation that the effect of rotation frequency on the emulsification is governed only by the average drop size and not by the full shape of its distribution. We also found similar observations for the influence of viscosity, volume fraction, and mixer size (not shown).

### Variation of the Mixer Radius

The emulsion is subsequently prepared with a smaller stator with a radius of *R*_mixer_ = 0.8 cm. As previously described, the emulsion is examined at successive rotation speeds, and the mean droplet diameters are shown as blue dots in Figure 5. Also, here the mean droplet diameter scales as the inverse of the rotation speed. The figure further shows that for a given rotation speed, the smaller mixer radius produces larger droplets.

As a first guess, one could assume that breakup occurs when inertial forces are balanced by viscous forces. The ratio of these forces corresponds to the Reynolds number *Re* = ρ_c_ω*R*_mixer_⟨*d*⟩/η_c_, where ρ_c_ is the continuous phase density and ω*R*_mixer_ is the speed at the tip of the blades. Thus, the balance between the inertial and viscous forces leads to ⟨*d*⟩ ∼ η_c_/ρω*R*_mixer_. This scaling is coherent with the experiments as the droplet diameter decreases with the rotation speed and the mixer radius.

### Impact of the Continuous Phase Viscosity

To further evaluate the previous scaling, we varied the viscosity, η_c_, of the continuous phase. To this end, we dissolved 3 wt % of SDS either in a water solution (η_c_ = 1 mPa s) or in a water/glycerol mixture (η_c_ = 6 or 11 mPa s with 50 or 60 wt % of glycerol, respectively). The emulsions were then prepared with a mixer with a radius of *R*_mixer_ = 0.8 cm.

**Figure 7:**
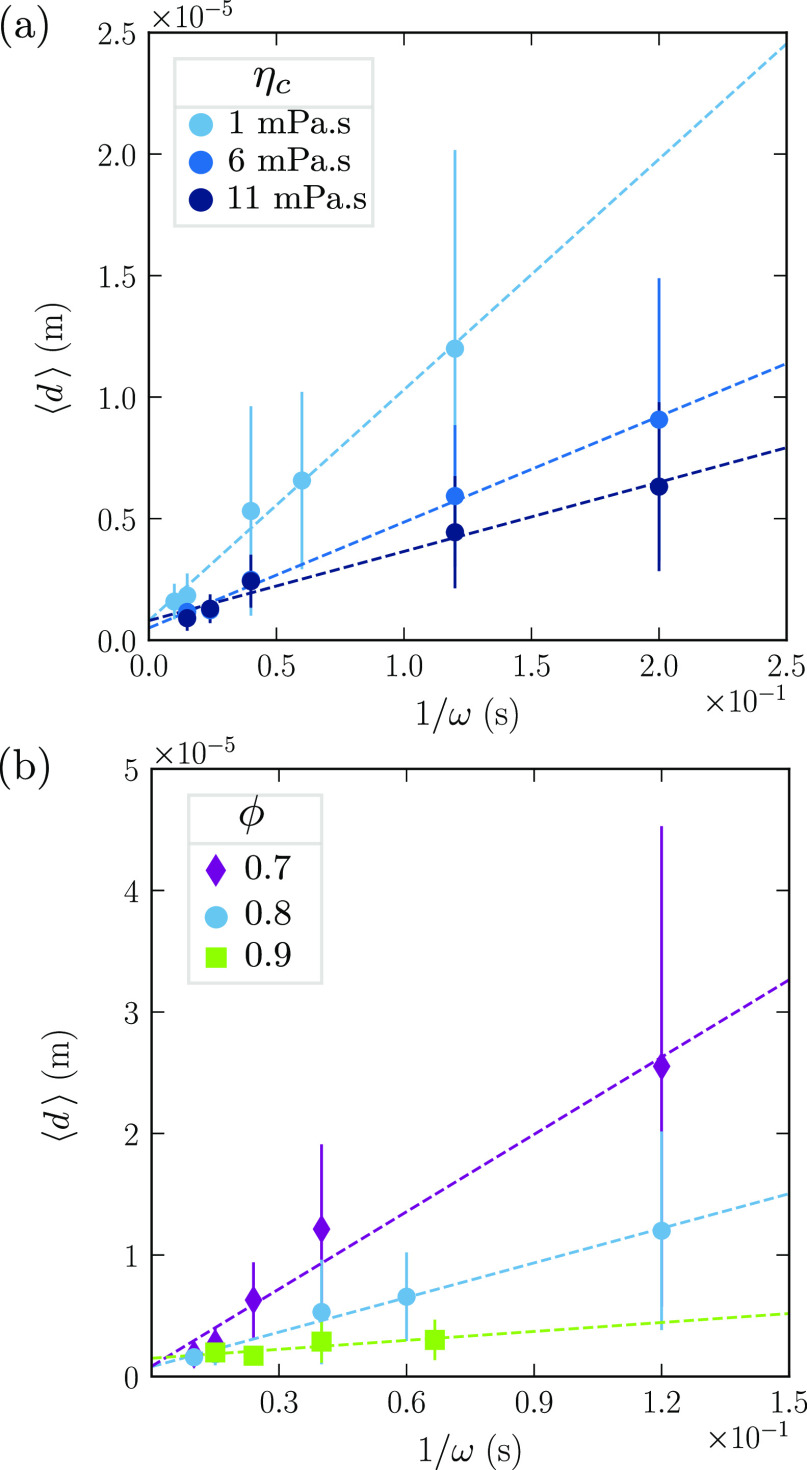
(a) By means of glycerol, the emulsions are prepared with varying continuous phase viscosities η_c_ = 1, 6, and 11 mPa s (from light to dark blue). The droplet size decreases with η_c_. (b) Mean droplet diameter ⟨*d*⟩ as a function of the inverse of the rotation speed ω for volume fraction ϕ = 0.7 (purple diamonds), 0.8 (blue dots), and 0.9 (green squares). The emulsions are prepared with a mixer of radius *R*_mixer_ = 0.8 cm and a continuous phase of viscosity η_c_ = 1 mPa s. Again, vertical bars indicate the standard deviation.

For each continuous phase viscosity, we measured the mean droplet diameter at successive rotation speeds. Figure 7a shows the mean droplet diameter as a function of the inverse of the rotation speed for η_c_ = 1, 6, and 11 mPa s (from light to dark blue). For a given rotation speed, the droplet size decreases with the viscosity. Hence, higher viscous forces result in smaller droplets. This trend contradicts our initial guess that the Reynolds number might be the relevant scale in droplet breakup.

As an alternative hypothesis for the breakup, we now concentrate on the surface tension forces compared to viscous stresses. The viscous stresses can either be modulated by the viscosity of the continuous phase or as an effective viscosity varied by different volume fractions for the dispersed phase.

### Variation of the Volume Fraction

To investigate the impact of the volume fraction, we disperse 70, 80, or 90 wt % of oil in the continuous phase. As previously reported, the total amount of oil is added to the aqueous phase at a low rotation speed. The latter is then increased, while samples of the emulsion are collected. Figure 7b shows the mean droplet diameter as a function of the inverse of the rotation speed for a varying volume fraction ϕ. The emulsions are prepared with a mixer of radius *R*_mixer_ = 0.8 cm and a continuous phase of viscosity η_c_ = 1 mPa s.

For a given rotation speed, the droplet size decreases with the volume fraction. Indeed, if a larger volume of oil is dispersed, then the effective viscosity increases. Thus, higher viscous forces are exerted, and the droplets are smaller.

As viscous forces have a great impact on the size, one can wonder if the droplet breakup could result from a local mechanism governed by a balance between viscous and capillary forces.

### Capillary Number Scaling

The process that breaks larger droplets into smaller droplets has to be against the surface tension. The stress required to deform a droplet of size *d* is γκ ∝ γ/*d*, where κ is its curvature. If the mechanism of droplet breakup comes from viscous shear stresses, which are the largest when the emulsion passes through the stirrer’s gap of size *L*, then these stresses scale as |η∇**v**|∝ ηω*R*_mixer_/*L*. Their ratio forms the capillary number
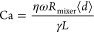
and droplets break in the given shear stress as long as Ca > 1, until they are small enough such that the surface tension can keep them in shape. We therefore expect the average droplet size to scale as
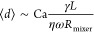
5

### Interfacial Tension Measurements

Measuring the interfacial tension between the oil and the continuous phase is not straightforward, since it depends on the concentration of the surfactant SDS, which changes during the emulsification process. The surfactants initially present in the continuous phase accumulate on the surfaces of the droplets, thereby establishing a dynamic equilibrium that relates the SDS concentrations on the surfaces to those in the bulk.

**Figure 8:**
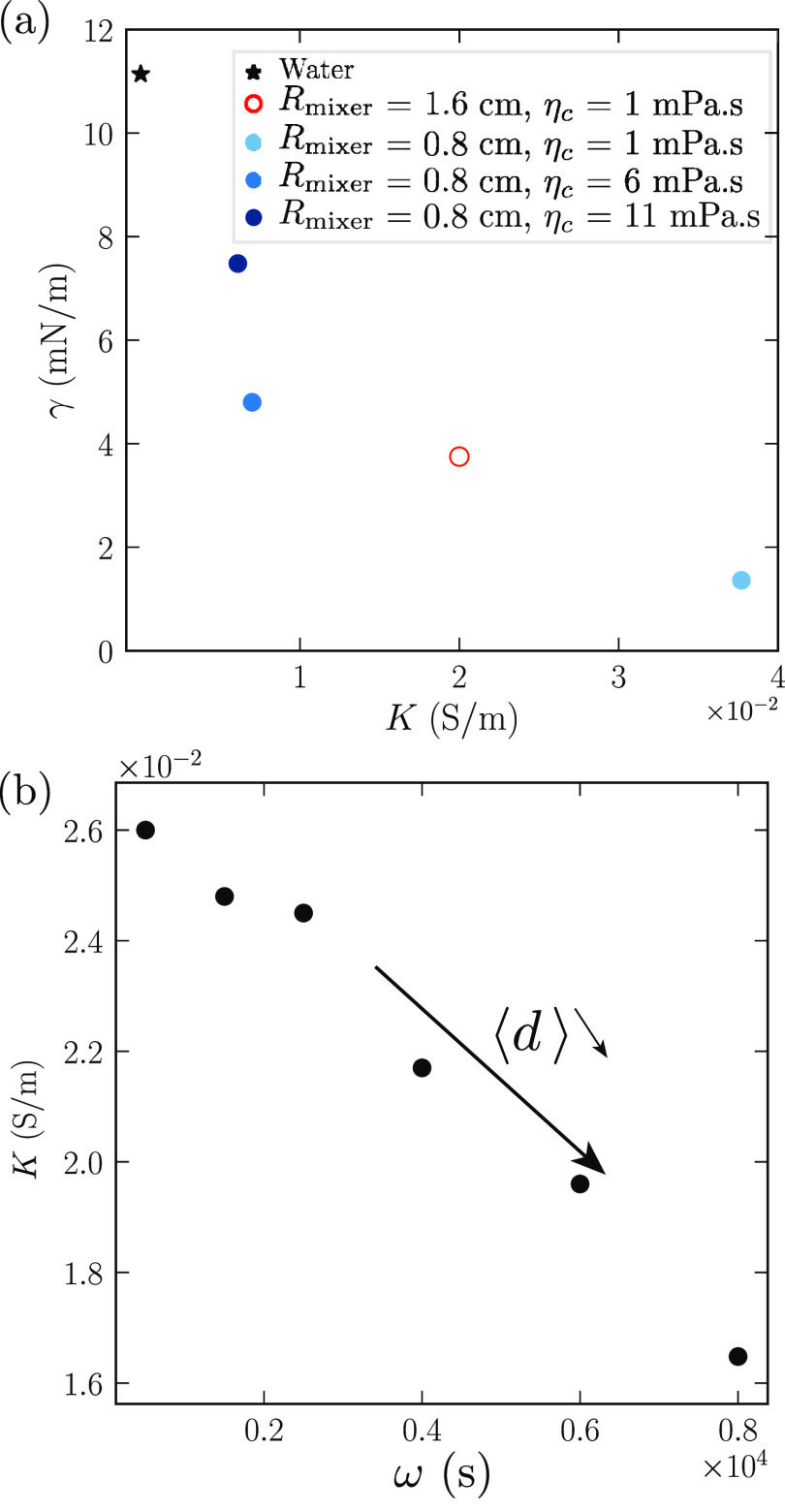
(a) Interfacial tension γ as a function of the conductivity of the emulsion. The tension is measured with the inverted pendant drop method using an oil drop immersed in a water/SDS solution mimicking the continuous phase of the emulsion. (b) Conductivity *K* of the emulsion as a function of rotation speeds. The emulsion has a volume fraction ϕ = 0.65, with 1 wt % of SDS, generated in a mixer of radius 0.8 cm.

The sodium ions from the SDS make the solution conduct, resulting in a monotonically increasing relationship between them. We can therefore conclude on the bulk SDS concentration by measuring the conductivity of the emulsion. Vice versa, if we happen to measure the same conductivity in a homogeneous solution of SDS as in a given emulsion, then we know they have the same bulk concentrations of SDS. This trick allows us to determine the interfacial tension of the droplets in the emulsion: we measure instead the tension of a macroscopic oil drop, deformed under the effect of gravity, by the so-called “inverted pendant drop method” in a homogeneous environment of SDS whose conductivity matches that of the emulsion. The result is shown in Figure 8a. As expected, the tension decreases with increasing conductivity from γ = 11 mN/m for *K* = 0.05 μS/cm (pure water) to γ = 1.4 mN/m for *K* = 377 μS/cm.

As the rotation speed increases, the droplets become smaller; thus, more and more surfactants leave the bulk to cover the interfaces, resulting in a decrease of the emulsion conductivity. Figure 8b shows this effect: the conductivity *K*, being directly related to the SDS concentration, is found to decrease as a function of rotation speed ω.

### Scaling

**Figure 9:**
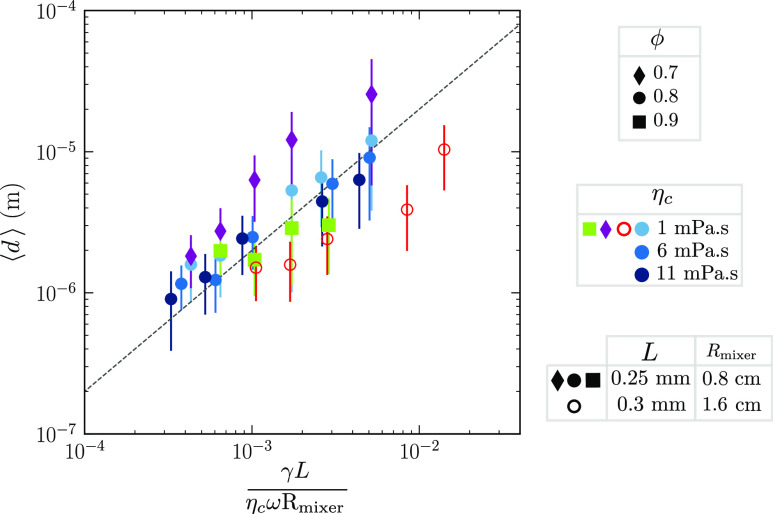
Rescaling of the droplet sizes in terms of the capillary number. The liquid parameters vary according to the legend. The dashed line is a guide to the eye to show linear scaling. Vertical offsets from the dashed line mean that the capillary number in eq 5 is not unity.

Using the above measurements of the surface tension, we can now check the role of the capillary number in droplet creation. In Figure 9 we plot the mean droplet diameter against the length scale given by the capillary number and the other parameters, eq 5.

We observe that linear scaling is found as expected, but it does not occur precisely at Ca ∼ 1. Instead, the breakup is shifted to higher capillary numbers for less concentrated suspensions and to lower capillary numbers for larger mixer radii. At the moment, we have no simple scaling argument for these deviations; we report them simply as an observation.

## Conclusions

The droplet size distribution of dense castor oil-in-water emulsions stabilized by the SDS surfactant was studied in the absence of droplet coalescence. It was found that the droplet size greatly varies with the rotation speed, the mixer geometry, and the continuous phase viscosity. The droplet size distribution was found to depend mainly on the average droplet size, not so much on changing its shape. Furthermore, it is somewhat more accurately described by a log-normal distribution rather than by a Gamma distribution; the former is expected for the breakup of droplets in a turbulent flow, and the latter suggests the formation of ligaments that subsequently break up in the flow. It would therefore be interesting to look into the details of the drop formation process, e.g., with numerical simulations.^31^ A simple scaling for the mean droplet diameter was derived without making strong assumptions about the complex turbulent flow. The mean droplet diameter was found to scale linearly in the length scale that relates surface tension to shear stresses. The precise value of the capillary number, however, still depends on the other material parameters of the emulsion. As such mixers are commonly used for emulsion manufacturing, the results here should be useful for controlling the rheology of emulsions for food, cosmetics, and various industrial applications. In addition, their stability, adhesive properties,^32^ and elasticity^33^ are also controlled by the drop size and volume fraction.

## Notes

After finishing the current paper, we were made aware of the existence of interesting work on the droplet size distribution resulting from individual breakups via a master equation.^34,35^
